# Challenges of providing healthcare worker education and training in protracted conflict: a focus on non-government controlled areas in north west Syria

**DOI:** 10.1186/s13031-020-00287-9

**Published:** 2020-07-08

**Authors:** Yamama Bdaiwi, Diana Rayes, Ammar Sabouni, Lina Murad, Fouad Fouad, Waseem Zakaria, Mahmoud Hariri, Abdelkarim Ekzayez, Ahmad Tarakji, Aula Abbara

**Affiliations:** 1 University of Oxford, Oxford, UK; 2 Syria Public Health Network, London, UK; 3 School of Public Health, University of California, Berkeley, CA USA; 4 Academic Primary Care, Hull York Medical School, Hull, UK; 5 Syrian American Medical Society, Washington DC, USA; 6 American University of Beirut, Beirut, Lebanon; 7 Syrian Board of Medical Specialties, Gaziantep, Turkey; 8 Conflict and Health, King’s College London, London, UK; 9 Department of Infection, Imperial College, London, UK

**Keywords:** Syria, Conflict, Education, Health professionals, Doctors, Nurses

## Abstract

Without healthcare workers (HCWs), health and humanitarian provision in Syria cannot be sustained either now or in the post-conflict phase. The protracted conflict has led to the exodus of more than 70% of the healthcare workforce. Those remaining work in dangerous conditions with insufficient resources and a healthcare system that has been decimated by protracted conflict. For many HCWs, particularly those in non-government-controlled areas (NGCAs) of Syria, undergraduate education and postgraduate training has been interrupted with few opportunities to continue. In this manuscript, we explore initiatives present in north west Syria at both undergraduate and postgraduate level for physician and non-physician HCWs. Conclusion: Challenges to HCW education in north west Syria can be broadly divided into 1. Organisational (local healthcare leadership and governance, coordination and collaboration between stakeholders, competition between stakeholders and insufficient funding.) 2. Programmatic (lack of accreditation or recognition of qualifications, insufficient physical space for teaching, exodus of faculty affecting teaching and training, prioritisation of physicians over non-physicians, informally trained healthcare workers.) 3. Healthcare system related (politicisation of healthcare system, changing healthcare needs of the population, ongoing attacks on healthcare.) Locally implementable strategies including dedicated funding are key to supporting retention of HCWs and return during post-conflict reconstruction.

## Background

Health workforce planning during conflict and in the post-conflict phase is essential to ensuring sufficient supply of healthcare workers (HCWs) of the right cadres and skills to meet the needs of the healthcare system [1]. The education and training of HCWs is fundamental to this however it is often neglected or adversely affected by political instability and conflict, particularly those which are complex and protracted such as in Syria. Syria’s conflict began with peaceful uprisings in March 2011 but by mid-2012 escalated into a conflict which has devastated its health system and eroded its healthcare workforce [2].

As of April 2020, more than 923 healthcare workers (HCWs) have been killed (mostly by the government of Syria and its allies) and more than 70% of HCWs have been forced to flee due to violence [3–5]. This has left those who remain working in an understaffed and underfunded healthcare system which is increasingly fragmented and politicised due to the prolonged conflict. The insufficient numbers of senior faculty has affected training, leadership and governance [5, 6] leaving undergraduates and junior postgraduate staff with inadequate training or mentorship and under conditions which require them to work beyond their training or expertise [5, 6]. The political complexities and ongoing violence with the competing needs of health and humanitarian priorities has also affected healthcare workforce planning with potential consequences during post-conflict reconstruction [7].

Healthcare needs of the population increase and change during protracted conflict due to violence, interruption of vaccination campaigns, effects on essential services and poor access to healthcare [2]. This occurs alongside the negative effects on the education and training of HCWs which affect the quantity, skills, distribution and quality of HCWs who enter the workforce [7, 8]. In Syria, the conflict has exacerbated existing pre-conflict geographical inequalities in healthcare access; Aleppo, Homs, Idlib, Dera’a, Rural Damascus and Deir ez-Zor have the lowest numbers of doctors per population [9]. Poor healthcare planning and ongoing violence have led to uncontrolled and largely unregulated expansion of private providers contributing to poorly planned, uneven distribution of health and medical services among geographical regions [10]. A population based survey that was performed in government and non-government controlled areas by the Syrian Centre for Policy Research noted that 31% of the population lived in areas where HCWs were insufficient and 27% live in areas where HCWs are completely absent [5].

There are no accurate estimates of the number, distribution and specialities of Syrian HCWs who remain in Syria and population mobility is such that estimates are quickly out of date. In 2018, the World Bank estimated the number of doctors per 1000 population in Syria to be 0.3 with a significant decrease from 2010 when it was 1.5 [9]. For nurses and midwives, the estimated decrease between 2010 and 2018 is from 1.4 to 0.6 per 1000 population [9]. In north west Syria, an area which has seen large scale population movements and increasing violence has an upper estimate of 1000 doctors (615 are hospitalists) for its 4.17 million population; there are up to 358 midwives, 1693 nurses and 709 community health workers [11]. It is important to note, however, that these estimates may be an overrepresentation of actual HCWs that exist given the lack of official registration and risk of double-counting HCWs who work in multiple facilities.

Healthcare workforce management in Syria is challenged by the fragmented healthcare system which has different health and political leadership across the country [2, 8]. Broadly, this includes areas under government control, north east Syria (under de facto Kurdish control), north west Syria (under opposition control) and areas in northern Syria which are under Turkish control [12]. This affects national as well as regional healthcare workforce planning and affects the education and training of undergraduate HCWs across the country but particularly those in areas outside of government control with some students who oppose the government in these areas intimidated or arrested [13, 14]. Areas outside of government control are also affected by an absence of universities whose degrees are accredited or recognised by international bodies [15].

Due to particular challenges arising in north west Syria with regards to the healthcare system, healthcare workforce and the potential to highlight illustrative factors relevant to HCW education and training during conflict, we have focused on this area [16]. During the course of the conflict, this area has developed a complex healthcare system where Syrian-led initiatives, Syrian non-governmental organisations (NGOs,) international NGOs and international organisations (e.g. WHO, UN) have provided cross-border health and humanitarian care; this has been mostly coordinated through the WHO-led Health Cluster in Gaziantep, Turkey [17]. Some of these organisations have also provided education or training to support the healthcare workforce on whom they draw on for staffing projects and healthcare facilities in north west Syria [17]. As of May 2020, it describes an area which includes Idlib, north west Hama, northern Aleppo and north eastern Lattakia governorates [18]. This area has seen a further escalation of violence in February 2019 and then again between December 2019 and March 2020 when almost 1 million civilians were forcibly displaced from their homes, including many HCWs [19].

Access to undergraduate HCW education and postgraduate training differs across geographical parts of Syria, particularly given the varying effects of the conflict across the country, the presence of an academic workforce, governing bodies, opportunities for continued training, accreditation, leadership, and specialization. Data from inside Syria is sparse as the situation has been rapidly changing with limited information about what undergraduate education or postgraduate training is available to HCWs in different areas in Syria. This is particularly the case for areas outside of government control, where volatile conditions and poor access for humanitarian agencies and researchers limits the ability to gather reliable, real-time information [5]. Therefore, in this manuscript, we explore current initiatives present in the north west of Syria at both the undergraduate and postgraduate level for physician and non-physician HCWs and the challenges faced in providing undergraduate education and postgraduate training during the conflict. This is with the aim of exploring the current provisions of HCW education and training and providing some comparisons to other conflict or post-conflict settings.

We conducted a desk-based literature review of available academic and grey literature which explore the undergraduate education and postgraduate training of healthcare workers including both formal and informal initiatives. We searched the websites of NGOs (non-governmental organisations) which are known to provide education opportunities to HCWs. We supplemented this with brief interviews to solicit clarifications from relevant stakeholders based within Syria and neighbouring countries to ensure that available material was up to date and to supplement what was found through the literature search. We also used notes from a meeting in Gaziantep where Syrian and international NGOs met to discuss HCW education in the summer of 2018. The interviews were not conducted as a formal qualitative research study; as such ethics review was not indicated.

For the purposes of this manuscript, the term non-physician HCWs is used to describe allied health professionals including physiotherapists, nurses, specialist nurses (e.g. neonatal, dialysis,) pharmacists, midwives, dentists, paramedics and emergency or anaesthetic technicians; the term physician HCW refers to doctors. We have used ‘education’ to refer to undergraduate education and ‘training’ to refer to postgraduate or speciality training for both physician and non-physician HCWs. The activities of the main public sector, private sector, NGOs and international organisations which support undergraduate and postgraduate HCW education and training are summarised in Table 1.

**Table 1:** This table summarizes the main activities of the public sector, private sector, NGOs and international organisations which support undergraduate and postgraduate health HCW education

	Main Undergraduate Actors	Explanation	Main Postgraduate Actors	Explanation
Public Sector	Free Aleppo University	Courses in Medicine, Dentistry, Pharmacy, Nursing available	Syrian Board of Medical Specialties	Provides post-graduate training programs to physicians; aim is to support non-physician training in future. Activities impeded by lack of funding, recognition and accreditation.
	Idlib University			
Private Sector	Various providers e.g. Al-Hayat University of Medical Sciences, North Syria Private University, Al-Shamal Private University	Courses in Medicine, Nursing provided. Poor regulation	No information available	No information available
Non-governmental organisations	Several including: UOSSM, SEMA, SAMS, HIHFAD	Mainly non-physician courses.	Several including: UOSSM, SEMA, SAMS, HIHFAD, Syria Relief, SRD	Mostly short course updates in person or via tele-education. Length varies from 2 to 3 days to longer programs. Includes training the trainers programs.
		Different training models including conversion courses and undergraduate courses. Unregulated.		
International Organisations	Nil	Nil	WHO. UNFPA	Mainly CPD courses and updates e.g. on infection prevention and control or mhGAP

*Abbreviations*: *UOSSM* Union of Syrian Medical Societies, *SEMA* Syrian Expatriate Medical Association, *SAMS* Syrian American Medical Society, *HIHFAD* Hand in Hand for Aid and Development, *SRD* Syria Relief and Development, *mhGAP* mental health Gap Action Program

### Undergraduate education

#### Overview

**Fig. 1:**
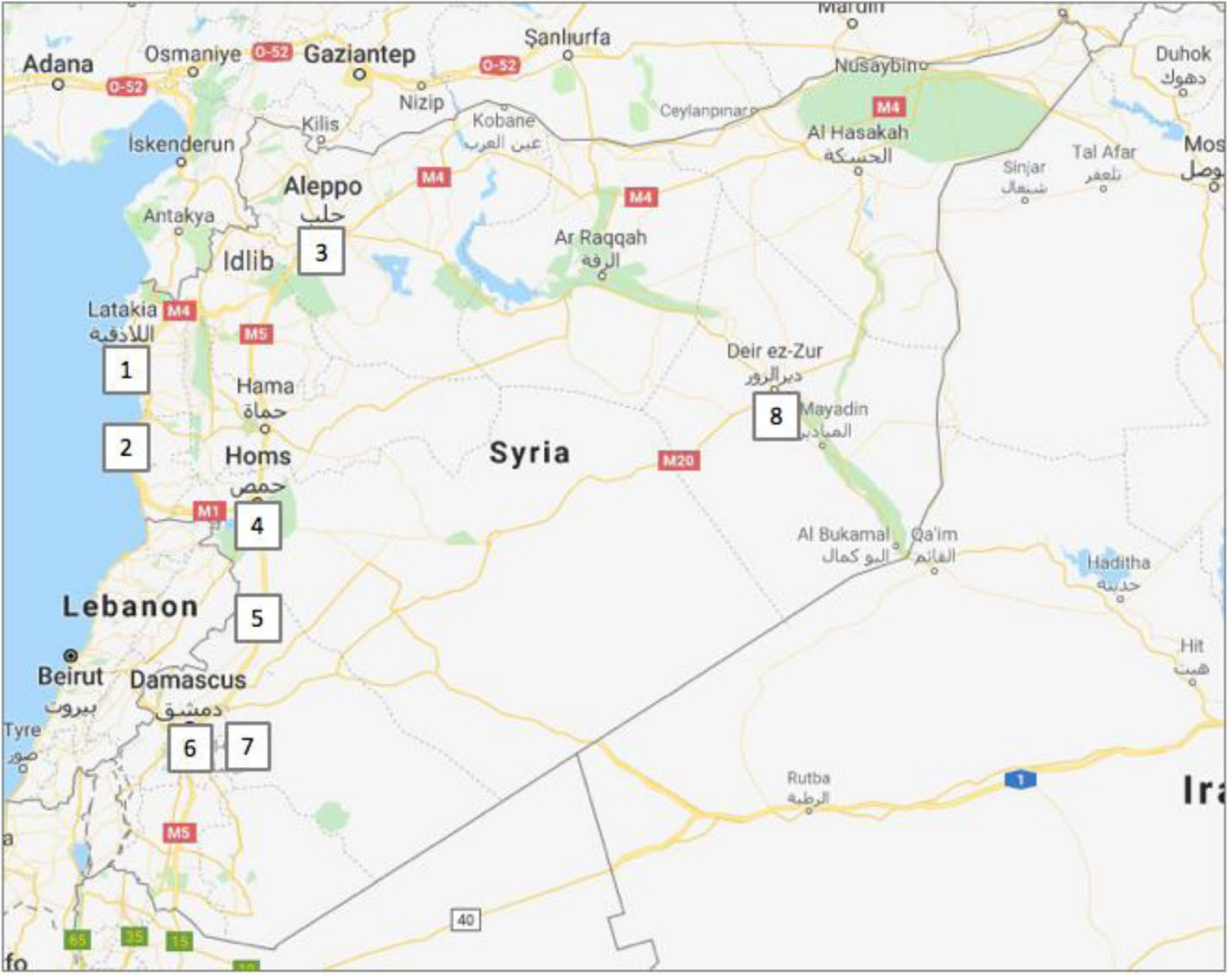
The distribution of medical schools in Syria before the war. 1. Tishreen University (Latakkia) 2. Al-Andalus University for Medical Sciences (Tartous.) 3. University of Aleppo 4. Al-Baath University (Homs) 5. University of Qalamoon (Deir Aityah) 6. Damascus University 7. Syrian Private University (Damascus) 8. Al-Furat University (Deir ez-Zor.)

Before the conflict, Syria had five public universities with Faculties of Medicine: Damascus University, University of Aleppo, Al-Baath University (Homs), Al-Furat University (Deir ez-Zor), and Tishreen University (Latakia) [20]. (see Fig. 1) Studies were delivered in Arabic. The structure of courses include basic medical sciences during the first 3 years with clinical modules in years 4 and 5 and clinical rotations in year 6 [21]. Aleppo University was the main university in the north west and was the second largest university in Syria. It had a number of Faculties including medicine, pharmacy, dentistry and nursing. The university ran six hospitals in Aleppo including Aleppo University Hospital, Aleppo University Cardiovascular Surgical Centre, Surgical Ambulance Hospital, Obstetrics and Gynaecology Hospital, Oral and Maxillofacial Surgical Centre and Al-Kindi Hospital.

Universities and undergraduate students have not been spared the effects of the conflict. The University of Aleppo was bombed on 15th January 2013, killing 82 people including students in an aerial attack [22]. Al-Kindi University Hospital (affiliated to Aleppo University) was destroyed by bombing [23]. The increased security threats, including bombardments, detention and torture, pushed many students to give up their studies and flee either out of the country or to NGCAs. Some of the most affected were those near the end of their degrees with little option to transfer to other universities inside Syria given security concerns including the risk of arrest by the GoS if they were thought to oppose the government or to have studied at ‘opposition institutions.’ [13, 14] For students and academic staff who remained in the NGCAs, some were able to continue their studies/lecturing in underground classrooms but others had to suspend their studies/academic career and contribute to humanitarian and healthcare provision to civilians affected by the conflict [13, 24]. Some were killed either during attacks on universities or during the course of the war [4].

As a result of the protracted conflict, targeting of healthcare and demand for trained HCWs in north west Syria, new faculties and institutes have been established in attempts to meet the education and training needs of physician and non-physician HCWs. Established facilities include three public faculties of medicine and three faculties of pharmacy at the Free Aleppo University (FAU), Idlib University and Shahba University; the latter was established in Dana to cover areas that are geographically far from Idlib University’s main campuses. There are six newly established healthcare institutes, three of which are still operating as of October 2019 (Termanin Institute, The Medical Sciences Academy in Qah, and Idlib University Institute) covering nursing and midwifery training, and three had been closed after repeated attacks (Kafr Sijneh institute, Birnas Institute, and Maarat Alnuman Institute). (personal communication) None currently have official recognition or accreditation for their degrees.

The demand from potential students and from potential employers has resulted in an economy around HCW education. This led to the founding of two private universities in north west Syria: Ebla University which was operating until 2018 but closed after repeated attacks and the North Syria private university which is still operating. Other private universities provide health-related degrees and established either before or after the conflict; some have continued to function while others have closes as they struggled to meet required standards set [25]. In this section, we discuss public universities, private universities and NGO-led undergraduate initiatives available in north west Syria in more detail.

i. ***Public Universities in North West Syria:***

The main public universities in the north west are the FAU and the University of Idlib. The FAU was founded as an alternative to universities in GCAs in December 2015 by the Ministry of Higher Education of the Syrian Interim Government and has since been contested in northeast Idlib by the rival opposition government, the Syrian Salvation Government [25, 26]. They provide degrees in a number of subjects including medicine, engineering and mathematics, however, there is no formal recognition or accreditation for these outside of NGCAs [25]. It is estimated that there are 5200 students in the 2018/2019 academic year, enrolled with campuses across the NGCAs. In the 2018/19 academic year, a second public university called Shabha University (previously known as Nahda University) was established; it is associated with the Higher Education Council and has been established in buildings that previously belonged to the FAU. It is in Al-Dana and has branches in Sarmada and Atareb [25].

The University of Idlib is associated with the Syrian Salvation Government and was formed in late 2017 [25]. It uses the infrastructure and the buildings which previously belonged to the Idlib campus of the University of Aleppo. It currently has 13,553 students across 16 faculties including faculties of medicine and dentistry; numbers increase to 15,000 students once the medical and dental students in Maarat al-Numan are included [25]. The Faculty of Medicine is listed in the World Directory of Medical Education however it has yet to receive international recognition [27].

ii. ***Private Universities in north west Syria***

The war has contributed to the necessity for and a trade in the provision of private healthcare education and training in north west Syria. However, these are often poorly regulated with little standardization or governance and have been more challenging for the local health directorates to regulate. They charge approximately 1000 USD per year and some provide options for distance-learning in addition to on-campus training. Examples of some of these universities include: Al-Shamal Private University which was set up with the merger of ‘Oxford University of Syria’ and the University of Rumah; the ‘Oxford University of Syria’ was opened as a branch of the Yemeni Oxford University which is recognised by the Yemeni Ministry of Education and the Arab League [25]. Other private universities include Mari University (established in 2015 in Mersin;) Osmania University (established in Istanbul in 2016, is a branch of the University of Malaysia, and has recognition in Yemen;) Al-Hayat University of Medical Sciences (established in Maarat al-Numaan in 2016 with disciplines in nursing, midwifery, physiotherapy and anaesthesiology.) [28] Some of these universities faced internal administrative, financial and governance challenges which affected their credibility and caused some to close. In addition, the Syrian Salvation Government has tried to enforce registration, permits and affiliation with it driving some private universities to close their doors [25].

iii. ***Non-governmental organisation led undergraduate initiatives***

Due to insufficient supply of HCWs and increased demand as well as the recognised need to fill the gaps left by public and private universities, some diaspora NGOs, often in conjunction with international organisations or universities have set up both undergraduate and postgraduate training initiatives to bridge gaps. These include undergraduate and postgraduate training, short courses, CME (continuous medical education), skills-based training and is aimed at physician and non-physician HCWs. Many educational and training opportunities have been created in response to the operational needs of the NGOs as well as the health and humanitarian needs of the population. Though NGOs have provided in-service training in other conflict or post-conflict settings [7] the extent to which this has been required to meet the needs in north west Syria has been more extensive and more sustained. Some international NGOs e.g. *Medecins sans Frontieres* have recognised the need for structured training for its local HCWs and have set up an Academy for Healthcare in 2017 to support HCWs in areas where they work [29].

A full review of the courses provided by NGOs is beyond the scope of this article, however some examples are given here. The Syrian American Medical Society (SAMS,) a US-registered humanitarian organisation, continues to support two training programs for midwives at Al Salam Obstetric Centre in Idlib. One is a 3-year training program where undergraduate enrolment occurs after high school and the other is an 18-month conversion course taken by qualified postgraduate nurses; there are currently 30 students in the former who will graduate in February 2021 [30]. They also support nursing education which started as 6–9 month courses in Idlib, Homs and Deraa. In 2017, they received funding to develop a two-year undergraduate nursing program in Termanin in Idlib; there are currently 53 first- and second-year students in general nursing and 11 in public health. In December 2018, 46 students graduated from the SAMS nursing program [30]. Most recently, a collaboration between SAMS and the Idlib Health Directorate resulted in the successful examination of 38 student midwives in Maarat Al-Nu’man and Omar Bin Abdul-Aziz in Termanin [31]. A UK-registered NGO called Hand in Hand for Aid and Development trained 205 healthcare workers (doctors, nurses, nursing assistants, midwives) in 2017 as part of their livelihood program [28]. The Syrian Expatriate Medical Association (SEMA) provides training through its Academy of Health Sciences [32]; in 2018, SEMA trained 65 nurses, 43 paramedics and 20 physiotherapists through 2 year diplomas [32]. They also provide online lectures for the students.

Though these initiatives fill an important gap and can be responsive to local needs, the courses provided by NGOs are not accredited or recognised outside the area however they do provide students with skills and opportunities to work with NGOs in north west Syria. These initiatives may be poorly coordinated, donor or NGO driven which could lead to duplication or gaps with little opportunity to standardise or provide quality assurance. This is being addressed by the local health directorates to ensure fair access to potential students and improved coordination.

### Postgraduate training

Postgraduate speciality training for physicians is provided the Ministry of Higher Education or the Ministry of Health however, in opposition areas, this would fall under the remit of the local health directorates. To fill this gap, Syrian led initiatives have been established. The most prominent of these in north west Syria is SBOMS (Syrian Board of Medical Specialties) which was set up mid-2015 with the aim of providing certification for the completion of speciality training after review of applicants’ experience and success at standardised examinations. It is affiliated to the Ministry of Health of Syrian Interim Government (SIG) [18] and works in coordination with health directorates in Idlib, Aleppo, and Hama to expand postgraduate and speciality opportunities for HCWs inside Syria, based on projected healthcare system needs.

SBOMS was set up as an independent legal and financial identity but is yet to be registered. They have 23 scientific committees (consisting of specialists who remain in Syria and expatriate Syrian doctors in the diaspora) who provide support for post-graduate training, examination and certification in a number of specialties including internal medicine, general surgery, vascular surgery, paediatric surgery, orthopaedic surgery, urology, paediatrics, cardiothoracic, obstetrics and gynaecology, ENT, ophthalmology, anaesthesia and intensive care, maxillofacial surgery, neurosurgery, and psychiatry. Though SBOMS has initially focused on physicians, they plan to expand to non-physician postgraduate training. Training is between 4 years (e.g. internal medicine, paediatrics) and 6 years (e.g. neurosurgery). So far, they have supported the training of 423 residents. In 2018, SBOMS successfully collaborated with the health directorates to support haematology and oncology specialty training for doctors in these governorates [33].

#### Non-governmental organisation led initiatives

The need for short updates which are focused on building the capacity of HCWs to meet the immediate needs of the population and the NGOs’ operational strategies has led predominantly Syrian NGOs to provide short updates for qualified HCWs. Some have been funded through private funds whereas others have been provided with funding from international NGOs or international organisations. Most of the training has been delivered by Syrian expatriate NGOs (predominantly SAMS, SEMA, UOSSM, HIHAID, Syria Relief, Syrian Relief and Development) either in Syria (in training centers in Idlib or Bab Al-Hawa on the Syria-Turkey border,) in Turkey (in Gaziantep, Reyhanli or Yayladag) or via tele-education. Courses provided range between 2 and 3 day updates on particular topics e.g. intensive care, general practice, microbiology, paediatrics to longer postgraduate courses which lead to certification; these include training for midwives, healthcare assistances, nurses, anaesthetic and dialysis technicians [32, 34].

Other initiatives have utilised expatriate Syrian experts or international trainers to deliver sessions or have developed collaborations for tele-education e.g. with Yale University, the University of Albany in the US, however many of these have not been sustained [35, 36]. Other providers including WHO have focused on training Syrian HCWs in particular topics such as infection prevention and control, post-surgical infections and Mental Health Gap Action Programme (mhGAP) [37]. UNFPA has supported a midwifery capacity building program began started a training-the-trainers program over 6 month periods during 2017 and 2018 with three sessions in Gaziantep and coaching during and after, in addition to certificates issued by UNFPA to accredit the trainees to be trainers in Syria [38]. In September 2019, the Idlib Health Directorate celebrated the conclusion of the UNFPA reproductive health training program, awarding 100 female reproductive health trainees with certificates indicating the success of their training, supervised by the UNFPA and the Health Cluster [39].

Though numerous training courses for HCWs have been held, there has been limited coordination, standardization or quality control for the training provided. Idlib Health Directorate has tried to address this through the appointment of a focal point whose role is to coordinate and prioritize topics for training courses for HCWs from Idlib and to liaise with providers. (personal communication) Training opportunities have also recently been hampered by the escalation of attacks in the north west since February 2019 as well as greater difficulties for Syrian HCWs to obtain Turkish permits to cross the Syrian-Turkish border in order to attend training in southern Turkey over the last 4 years [40]. The opening of training centres in Idlib (one in 2017 and one in September 2019) may support further opportunities for the education of HCWs however the entry of foreign experts to Idlib via Turkey will be limited given ongoing security concerns and border closures due to the ongoing COVID-19 pandemic. As such, increasing use of tele-education is being put in place to deliver remote education.

#### Tele-education and distance learning

In Syria, tele-education has been used with mixed success with challenges including logistics and cost, consistent expertise outside of Syria, connectivity and, in the case of clinical skills, the benefits that in-person training would provide. Blended modes of learning e.g. tele-education with some in-person training or contact time if possible may be the best approach to ensure relevant theoretical and practical skills are introduced. Given current insufficient numbers of educators among the healthcare workforce in north west Syria, more efforts to capitalise on available technologies which support the education of HCWs is needed. Tele-education has been used in other conflict and post-conflict settings including Iraq, Gaza [41, 42], Bosnia and Herzegovina [43]; some of these have been collaborations with international universities e.g. Mayo clinic, Queen Mary University in London and have usually focused on a single topic e.g. burns care, intensive care education. Further work to provide sustained and evaluated courses is needed to meet the training needs of HCWs in north west Syria.

## Discussion

Despite the clear need for a skilled workforce of sufficient number and training to meet the current and future demands of north west Syria’s complex healthcare system, the provision of HCW education and training remains fragmented, politicised and uncoordinated. HCWs continue to face numerous obstacles, including ongoing interruptions to education and training, lack of access to advanced specialty training and professional development opportunities of sufficient quality and which are accredited and recognised outside of the area. Providing relevant opportunities can improve retention of HCWs and may support return of those who have been forced to flee, promoting long-term peacebuilding efforts [44]. As such, focused and realistic strategies which include key stakeholders and which are led and coordinated by local governing bodies (health directorates) could improve opportunities for HCW education and training in north west Syria. This could have positive consequences on the local healthcare system including the retention or return of HCWs, and the overall reconstruction of the Syrian healthcare system.

### Key challenges

Challenges to HCW education in north west Syria can be broadly divided into 1. Organisational (local healthcare leadership and governance, coordination and collaboration between stakeholders, competition between stakeholders and insufficient funding.) 2. Programmatic (lack of accreditation or recognition of qualifications, insufficient physical space for teaching, exodus of faculty affecting teaching and training, prioritisation of physicians over non-physicians, informally trained healthcare workers.) 3. Healthcare system related (politicisation of healthcare system, changing healthcare needs of the population, ongoing attacks on healthcare.)

Similar challenges have been highlighted in other conflict affected contexts including Iraq [45] and Gaza [46] where politics, economics and inability to recruit educators have been noted. In Gaza, an additional challenge highlighted is the disorganised post-graduate programs with limited continuing professional development; this has been cited to be a factor contributing to low morale among doctors [46].

**Fig. 2:**
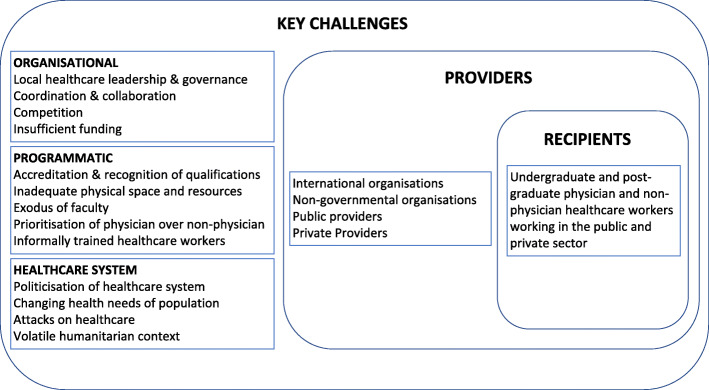
A framework which shows the key challenges which affect the providers and recipients of healthcare worker education and training

Figure 2 summarises key challenges in north west Syria and their relationship to providers and recipients of HCW education. Some of the key challenges are explored in more detail below.

### Local leadership and the role of diaspora humanitarian organisations

Educational governance in north west Syria has been challenging though public institutions and an increasing number of the NGO led initiatives have monitoring and evaluation programs in place [30, 32]. However, there remains a lack of oversight or a standardised approach with NGOs often answerable to their funders to a greater extent than local governing bodies. For private led initiatives, regulation has proven particularly challenging [25]. Idlib Health Directorate has led a number of initiatives to address issues surrounding educational governance and have developed a central database of the healthcare workforce including skill-mix and training gaps, coordinated capacity building initiatives across health facilities, identified physical spaces where training can occur and have supported programs which meet the needs of the healthcare workforce. SBOMS has taken a leading role in postgraduate training and certification (with support from diaspora Syrian HCWs) to provide standardisation to specialty training; for internal medicine postgraduate specialty training, a diaspora NGO (SAMS) provided a stipend and training for doctors as a financial incentive to allow them to work and provide much needed healthcare for the local population while training. However, similar initiatives and the work of SBOMS and the Idlib Health Directorate are met with funding constraints which affect sustainability and planning.

The funding shortfall for health and humanitarian provision to north west Syria is large with very limited financial resources available for HCW education and training. For example, donor funds have been redirected from local health directorates to the WHO to implement and oversee HCW educational programs, weakening the role and influence of local health directorates [47]. This can partly be addressed by a dedicated funding stream for HCW education and training and for funders to allow some project funds to be allocated to education and training.

### Accreditation and recognition

Though undergraduate degree programs are provided in north west Syria, they are unrecognised outside of the local area and the programs do not have accreditation. A number of attempts have been made to address this however none have so far been successful; this may present challenges for the graduates. A similar challenge occurred during the Balkan conflict with the consequences for the HCW both during and after the conflict [48]. After 1989, many Albanians lost their jobs leaving them without insurance; as a result, Albanian health professionals set up a parallel primary healthcare system. Albanian doctors and nurses, who were unable to study in their own language in Pristina University were trained through this parallel health system during the 1990s [48]. However, though the 600 doctors and 1200 nurses who graduated from this system may have had sufficient theoretical knowledge (clinical training was harder to obtain), this mode of training left a generation of Albanian doctors and nurses with unrecognised qualifications, subsequently affecting their ability to work [48]. As such, a similar scenario in north west Syria, could leave thousands of HCWs in north west Syria with certificates or degrees which are not recognised outside of the region limited their career prospects; this could affect retention of HCWs who may seek opportunities elsewhere though, conversely, it may support the retention of some HCWs who are unable to leave the local healthcare system as a result.

### Informally trained healthcare workers

A group of HCWs who have received little attention are those who have gained training in healthcare informally. These may be students who left their degrees due to the conflict but continued to work in hospitals or clinics or those who began providing healthcare to injured civilians and gained ‘on the job’ training particularly where there were shortages of trained HCWs. They are disadvantaged even compared to those whose degrees or qualifications are unrecognised or unaccredited.

Due to the protracted nature of the conflict, many, particularly those who are wanted by the government, may have left Syria to complete their studies elsewhere but others may have continued to work in the local health system gaining experience and attending courses providing by Syrian NGOs. According to UOSSM 2015’s hospital surveillance, 36% of the 696 nurses working in the north west fell into this category and there is concern as to what their role will be post-conflict as they have not participated in recognised training programs [49]. However, with the role of newly established institutes, the Idlib Health Directorate reports that this percentage is declining. (personal communication.) One of the roles of SBOMS has been to review this ‘on the job’ training in collaboration with the health directorates in north west Syria with a view to identifying those who can receive credit for this.

### Skill substation (task shifting)

Physicians in Syria are highly respected and are often community leaders; during the course of the Syrian conflict, many have established health or humanitarian NGOs or taken leadership positions. This, together with the more standardised pathways for physician training, may have contributed to greater opportunities for physician compared to non-physicians HCWs in north west Syria. Subsequently, the insufficient numbers of HCWs in the area, have led to the recognition that increased focus on non-physicians HCWs or on skill substitution (the transfer of tasks normally performed by doctors to other health professionals with different skills or levels of training) as potential solutions [50]. Skill substitution (formerly task shifting) has been discussed by the WHO and World Medical Association for some years and has an important future role in both high- and low-income countries as well as in humanitarian crises to reduce costs and meet the needs of the population [50].

In north west Syria, skill substitution has already occurred in some contexts; for example, specialised dialysis nurses had been taking the roles of renal specialists to oversee the estimated 500 dialysis patients managed by Syrian diaspora NGOs in the north west; this was done with the support of the single remaining renal physician and a team of expatriate renal physicians providing training and advice. (personal communication) However, as yet, the acceptability of skill substitution to Syrian patients amongst Syrian HCWs as one of the solutions has not been fully explored. This is particularly the case as proposed models which may be suited to other conflict affected settings, may not be as acceptable to the local population in Syria due to the heavily medicalised and specialist model of the health system before the war [10]. Locally acceptable solutions are therefore urgently required given the massive skill and number shortage of HCWs which is likely to persist for some years [7, 44].

### Political influences

**Fig. 3:**
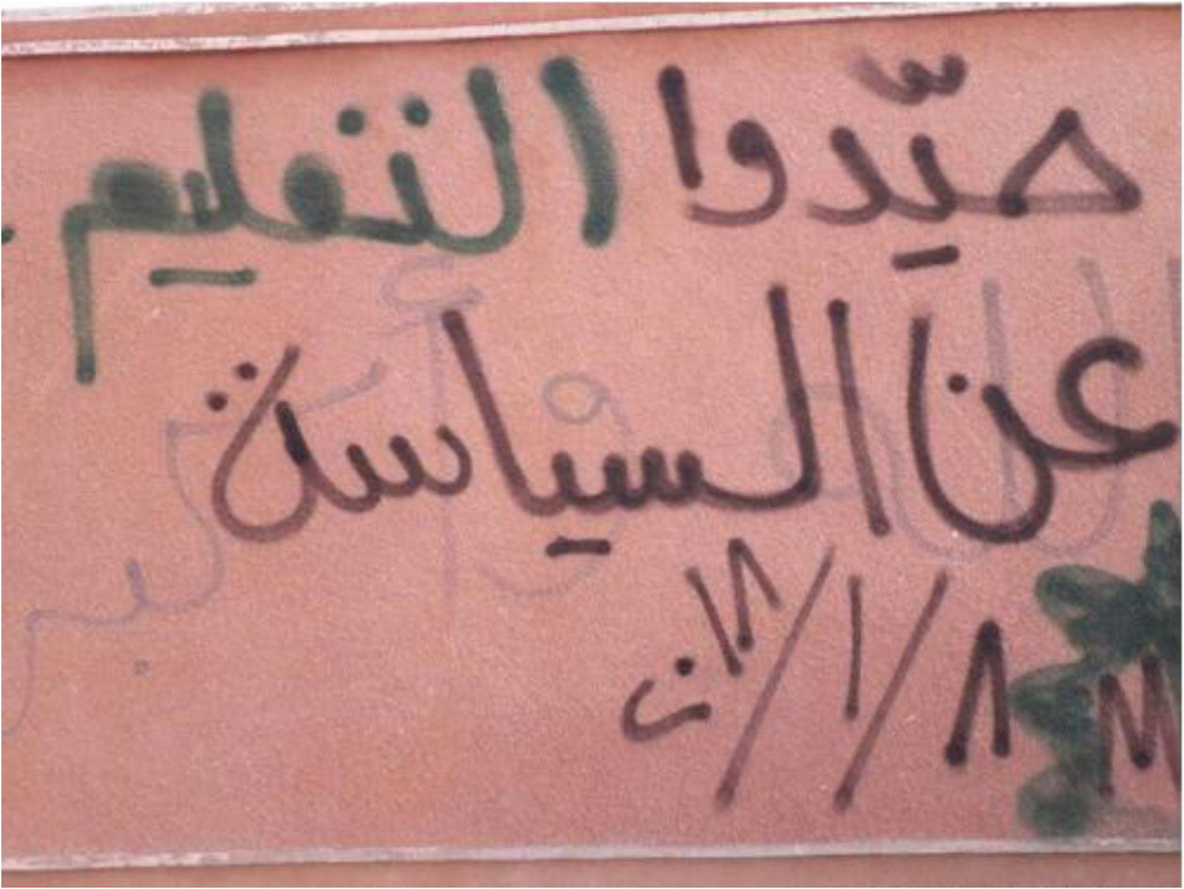
Free Aleppo University graffiti in Arabic which reads ‘Keep education separate from politics’ and is dated 8th January 2018. Photo Credit to Free Aleppo University

Local and international political developments have a number of effects on HCW education and training. Syria’s health system is increasingly fragmented and politicised; in north west Syria, there are shifts in groups who control the area and their influence over local institutions [26]. For example, when the Syrian Salvation Government took control of Idlib and parts of the western countryside of Aleppo, it insisted that local educational institutions be affiliated to it; this led some institutions to close and others to relocate. Classes were interrupted and students staged sit-ins and called for politics to remain separate from education [26]. (see Fig. 3). Political influences are noted in other conflict affected contexts in Gaza, Iraq and the Balkans [46, 48].

HCWs also face challenges crossing check points to attend training, examinations or to participate in educational activities in government controlled areas; reports of questioning and arbitrary arrests are widespread [14]. Any affiliation with a NGCA institution has been considered criminal by the government of Syria. As such, when the government of Syria reclaimed Aleppo, students burned their books and study materials to avoid being linked to one of the opposition-led universities which would result in arrest [14]. Students and faculty in government controlled areas would be stopped at university or hospital checkpoints, questioned and sometimes arrested without charge. These individuals were arrested by security forces that had set up offices within university campuses, hospitals, or by appointed student members of the Syrian Student Union. The offices of the Student Union were sometimes used as temporary detention centres for arrested students and faculty, and there have been documented instances of arrested HCW student and faculty being tortured in these offices before being taken to Security force centres. Some of those arrested later died under torture [51].

### Towards a shift in prioritising healthcare worker education and training

The response to HCW education and training in north west Syria has seen an important shift in the humanitarian system whereby local and diaspora led initiatives including from NGOs have identified critical gaps in HCW education and training and sought to meet them [30, 32, 34]. Though this has occurred in other conflict affected settings e.g. Gaza, Iraq, Balkans, the extent to which these organisations have responded to the needs in north west Syria and their sustained attempts to meet critical gaps and to replace previous educational institutions and accreditation bodies has been more extensive than seen in previous conflicts [41, 42, 48]. This may represent a shift change in the humanitarian system whereby HCW education (with regulation/ certification/ accreditation) is prioritised alongside other key sectoral needs, particularly for complex and protracted crises such as Syria. This could draw on the experience of international organisations or NGOs e.g. *Medicins sans Frontieres* [29] with lessons learned in Syria informing these discussions.

### Areas outside of north west Syria

While this manuscript focuses on north west Syria, students in Faculties of Medicine or other Health Sciences in other areas of Syria, including government-controlled areas have also suffered the effects of the conflict. Students in government-controlled areas who could safely remain were able to complete their studies with a change in the regulations. Students were allowed to fail up to 6 or even 8 classes of the 12 required [13]. Students from Al-Baath, Aleppo and al-Furat could attend classes at safer campuses with thousands of students able to transfer to Damascus or Latakia universities. After the National Hospital of Homs was destroyed, agreements were made to allow students to take clinical placements at private hospitals. Due to these exceptions, standardized final examinations were introduced for medicine, dentistry, pharmaceutical studies and nursing (as well as informatics engineering.) [13, 21]

There are important topics which influence any discussion around the training of HCWs in Syria which have not been explored in this manuscript. These include the changing health needs of Syria’s population where there is a high burden of non-communicable diseases, conflict-related disability and a traumatised population [52]; the severe psychosocial stresses experienced by the HCWs both first-hand and as secondary trauma [44, 52, 53]; and the weaponization and destruction of healthcare in Syria [5, 54, 55] which affects HCWs’ ability to focus on their own education and training needs. Table 2 summarizes some of the key messages from this manuscript together with broad recommendations for the future. These recommendations need to be developed further with on the ground actors to ensure they are locally practicable and, given resource constraints, prioritized.

**Table 2:** Key messages on healthcare worker education and training in north west Syria and broad recommendations to address challenges identified

Key Messages	
1.	Syria’s protracted conflict has decimated its health system and led to a profound shortage of healthcare workers of sufficient number and skills, exacerbating pre-existing geographical inequalities.
2.	Both physician and non-physician healthcare worker undergraduate and post-graduate training has been affected across the whole of Syria but the impact has been greatest in areas outside of government controlled, particularly in north west Syria.
3.	Free Aleppo University and Idlib University and Shabha University are the main public universities which provide undergraduate medical and pharmacy education in north west Syria however they lack sufficient funds and faculty to support quality and sustainability.
4.	There has been an unregulated growth in the number of private universities which provide undergraduate physician and non-physician healthcare worker education.
5.	Unusually in a humanitarian response, non-governmental organisations have stepped in to provide some undergraduate courses as well as shorter, postgraduate continuous medical education opportunities for healthcare workers however this has been uncoordinated and unsustained.
6.	There remains a gap in post-graduate physician training though organisations like the Syrian Board of Medical Specialties provide some opportunities though they are affected by poor funding and insufficient numbers of faculty with the specialties required.
7.	Challenges can be broadly divided into 1. Organisational 2. Programmatic and 3. Healthcare system related
**Broad Recommendations**	
1.	Develop a locally driven healthcare worker education strategy for north west Syria which is developed in close collaboration with key local and international stakeholders and which could lead to the formation of a consortium focused on healthcare worker education.
2.	Ensure dedicated funding streams for healthcare worker education which are multi-year and accessed through the consortium is the ideal but may be not be feasible; however, allowing funding as part of grants could allow dedicated funds for healthcare worker education.
3.	Develop relevant governance and regulatory structures which standardise the minimum quality of public and private educational establishments which deliver healthcare worker education.
4.	Develop strategic partnerships with international institutions which could support accredited and recognized courses for physician and non-physician healthcare workers in north west Syria. This could be supported with more developed tele-education interventions.
5.	Increase focus on non-physician healthcare workers and skill substitution (task shifting) is required to ensure a healthcare workforce of sufficient skill and specialty to meet gaps.
6.	Conduct regular reviews of initiatives which have or have not been effective (e.g. quality, cost) in providing physician and non-physician healthcare worker education is needed to inform future initiatives.
7.	Continue to advocate for the protection of all health workers and healthcare provision in conflict, which is essential for the continuation of work and training without fear of attack.

## Conclusion

Challenges faced in delivering HCW education and training will affect the numbers, skills and distribution of HCWs in north west Syria both now and in the post-conflict phase. Improved coordination by all stakeholders with a medium and longer-term strategy that is implementable in the current context is needed. This requires sufficient and sustained investment from multilateral organizations, such as the UN, and international donors. The situation in north west Syria shares some similarities but also important differences in terms of HCW education and training during conflict. As such, lessons can be learned from the Syrian context with opportunities to support earlier adoption of innovations e.g. tele-education, skill-substitution for both ongoing and future conflict-affected contexts and ensure robust leadership and governance. The need for this has been further underlined by the COVID-19 pandemic which has highlighted the need for a robust healthcare workforce and healthcare systems which can effectively meet the needs of the response. Lastly, we highlight that efforts in education and training for HCWs without protection as stipulated under International Humanitarian Law are futile.

## Data Availability

There is no extra available data other than that which is quoted and referenced in the text.

## Abbreviations

CME: Continuous Medical Education
FAU: Free Aleppo University
GCAs: Government-Controlled Areas
HCWs: Healthcare workers
NGCAs: Non-Government-Controlled areas
NGO: Non-Governmental Organisation
NW Syria: North West Syria
SAMS: Syrian American Medical Society
SEMA: Syrian Expatriate Medical Society
UNFPA: United Nations Population Fund
UOSSM: Union of Medical Care and Relief Organisations

## References

[1] World Health Organization. *Guide to Health Workforce Development in Post-Conflict Environments.* World Health Organization; 2005.

[2] Fouad FM, Sparrow A, Tarakji A (2017). Health workers and the weaponisation of health care in Syria: a preliminary inquiry for the lancet –American University of Beirut Commission on Syria. Lancet..

[3] Physicians for Human Rights. Medical Personnel are Targeted in Syria. https://phr.org/our-work/resources/medical-personnel-are-targeted-in-syria/. Published 2019.

[4] Physicians for Human Rights. A Map of Attacks on Health Care in Syria. Physicians for Human Rights. The Destruction of Hospitals – A Strategic Component in Regime Military Offensives. https://syriamap.phr.org/#/en/case-studies/5. Published 2019. Accessed 22 July 2019.

[5] Fouad FM, Sparrow A, Tarakji A, et al. Health workers and the weaponisation of health care in Syria: a preliminary inquiry for The Lancet American University of Beirut Commission on Syria. Heal Policy 2516 www.thelancet.com. 2017;390. 10.1016/S0140-6736(17)30741-9. 10.1016/S0140-6736(17)30741-9 28314568

[6] Ismail S et al. Strengthening Human Resources for Health: Integration of Refugees into Host Community Health Systems.; 2017. http://www.cmimarseille.org/highlights/strengthening-human-resources-health-integration-refugees-host-community-health-systems.

[7] Roome E, Raven J, Martineau T (2014). Human resource management in post-conflict health systems: review of research and knowledge gaps. Confl Health..

[8] Abbara A, Blanchet K, Fouad F, Sahloul Z, Coutts A WM. The effect of the conflict on Syrian health system and human resources for health - health in humanitarian crises Centre. World Heal Popul 2015;16(1):87–95. http://crises.lshtm.ac.uk/2016/04/20/aula-abbara-blanchet-karl-f-f-zaher-sahloul-adam-coutts-and-m-wasim-2015-the-effect-of-the-conflict-on-syriais-health-system-and-human-resources-for-health-world-health-populatio/. Accessed 14 July 2017.

[9] World Bank and UNHCR. The Mobility of Displaced Syrians An Economic and Social Analysis.; 2019. https://reliefweb.int/sites/reliefweb.int/files/resources/9781464814013.pdf. Accessed 22 July 2019.

[10] Kherallah M, Alahfez T, Sahloul Z, Eddin KD, Jamil G (2012). Health care in Syria before and during the crisis. Avicenna J Med.

[11] WHO. WHO Gaziantep Field Presence, Turkey (Medical Portable Ventilator Machine)-Availability of Health Equipment Monitored through HeRAMS Tool.; 2020. https://www.humanitarianresponse.info/sites/www.humanitarianresponse.info/files/documents/files/herams_1st_quarter_2020_v2.pdf. Accessed 30 Apr 2020.

[12] Abbara A, Rayes D, Fahham O, et al. Coronavirus 2019 and health systems affected by protracted conflict: the case of Syria. Int J Infect Dis. May 2020. 10.1016/j.ijid.2020.05.003. 10.1016/j.ijid.2020.05.003 PMC7205638 32389845

[13] Al-Fanar Reporting Team. The Difficulty of Studying Medicine in Times of War — Syria Deeply. News Deeply. https://www.newsdeeply.com/syria/articles/2017/10/30/the-difficulty-of-studying-medicine-in-times-of-war. Published 2017. Accessed 22 July 2019.

[14] Students of Opposition School System ‘Start from Zero’ in Government-Held Aleppo City - Syria Direct.; 2017. https://syriadirect.org/news/students-of-opposition-school-system-‘start-from-zero’-in-government-held-aleppo-city/?fbclid=IwAR2wVlebk1yQM23_47s3unah_NgAn_uZMXOfYaUEDkt6YlHuyN5rFxaT9UE. Accessed 14 Oct 2019.

[15] FAQs. The World Federation for Medical Education. https://wfme.org/accreditation/faqs/. Accessed 9 May 2020.

[16] REACH. North-West Syria Population Overview: November 2018 - Syrian Arab Republic | ReliefWeb.; 2018. https://reliefweb.int/report/syrian-arab-republic/north-west-syria-population-overview-november-2018. Accessed 14 Oct 2019.

[17] Duclos D, Ekzayez A, Ghaddar F, Checchi F, Blanchet K (2019). Localisation and cross-border assistance to deliver humanitarian health services in north-West Syria: a qualitative inquiry for the lancet-AUB Commission on Syria. Confl Health..

[18] UN. Risk grows of ‘catastrophic humanitarian fallout’ in Syria’s Idlib, where 3 million are trapped: top UN officials urge unity in Security Council | UN News. https://news.un.org/en/story/2019/05/1038681. Accessed 23 July 2019.

[19] Abbara A, Rayes D, Khalil M, Kewara M, Tarakji A. Humanitarian catastrophe for civilians in Northwest Syria. BMJ. 2020;368. 10.1136/bmj.m451. 10.1136/bmj.m451 32029428

[20] Geneva Foundation for Medical Education and Research. Medical schools, governments, ministries, medical associations : Syria. https://www.gfmer.ch/Medical_search/Countries/Syria.htm. Published 2019. Accessed 22 July 2019.

[21] Sawaf B, Abbas F, Idris A, Al Saadi T, Ibrahim N (2018). Specialty preference and intentions to study abroad of Syrian medical students during the crisis. BMC Med Educ.

[22] Simpson, C. This Is What It Looks Like When Syria Bombs a University - The Atlantic. https://www.theatlantic.com/international/archive/2013/01/syria-bombs-aleppo-university/319456/. Accessed 22 July 2019.

[23] Aleppo’s largest hospital in rebel-held area is “destroyed” | World News | Sky News. https://news.sky.com/story/russian-planes-destroy-syrias-cave-hospital-10604281. Published 2016. Accessed 14 Oct 2019.

[24] Abbara A, Orcutt M, Gabbar O (2015). Syria’s lost generation of doctors. BMJ..

[25] Abdel Nour, Nour; Shahadeh H. Universities of north Syria: Future hindered by crises – Enab Baladi. Enab Baladi. https://english.enabbaladi.net/archives/2019/07/universities-of-north-syria-future-hindered-by-crises/?fbclid=IwAR2_yQJMxVtBAJSVTox3RwUjZGdmz_aSrRU7V5QjEeZLAZXyq7jmLYSAiIA. Published 2019. Accessed 22 July 2019.

[26] Al Nodal Walid NM. A power struggle over education emerges between rival opposition governments in Idlib province - Syria Direct. https://syriadirect.org/news/a-power-struggle-over-education-emerges-between-rival-opposition-governments-in-idlib-province/. Published 2018. Accessed 22 July 2019.

[27] School Detail. World Directory of Medical Schools. https://search.wdoms.org/home/SchoolDetail/F0005502. Accessed 22 July 2019.

[28] Terkawi A, Bakri B, Alsadek A (2019). Child and adolescent health in northwestern Syria: findings from healthy-Syria 2017 study. Avicenna J Med..

[29] MSF Academy for Healthcare | MSF. https://www.msf.org/academy. Accessed 2 May 2020.

[30] SAMS. SAMS Annual Report *2018*.; 2019. https://www.sams-usa.net/wp-content/uploads/2019/06/SAMS-Annual-Report-2018-V5.pdf.

[31] Conducting a colliculum exam for students of midwifery institutes | Idlib Health Directorate. https://ihd-sy.org/en/2019/10/15/conducting-a-colliculum-exam-for-students-of-midwifery-institutes/. Accessed 28 Oct 2019.

[32] Medical Education and Training – الرابطة الطبية للمغتربين السوريين. https://www.sema-sy.org/en/medical-education-and-training/. Accessed 22 July 2019.

[33] The Syrian Board of Medical Specialties (SBOMS) announces the general rating of students for admission to blood and tumors specialties. – موقع مديرية صحة حماه. https://hama-hd.com/en/the-syrian-board-of-medical-specialties-sboms-announces-the-general-rating-of-students-for-admission-to-blood-and-tumors-specialties/. Published 2018. Accessed 22 July 2019.

[34] Development H In H for a and. Hand in Hand for Development and Aid. Annual Report 2018. Publised 2018.

[35] Kaylin J. In the Midst of War, Future Syrian Doctors Trained with Help From Yale Faculty, Students | Yale School of Public Health. https://publichealth.yale.edu/article.aspx?id=13702. Published 2016. Accessed 22 July 22, 2019.

[36] TimesUnion. UAlbany online science courses for refugee Syria medical students. TimesUnion. https://www.timesunion.com/tuplus-local/article/Free-UAlbany-online-science-courses-for-refugee-9194192.php. Published 2016.

[37] WHO | mhGAP. Training creates more support for vulnerable people in Syria. *WHO*. 2015. https://www.who.int/mental_health/mhgap/syria_story/en/. Accessed 22 July 2019.

[38] Quality Midwifery Care in the Midst of Crisis: Midwifery Capacity Building Strategy for Northern Syria. 2017–2021.; 2017.

[39] The conclusion of the activity of medical education center to train Reproductive Health Providers. | Idlib Health Directorate. https://ihd-sy.org/en/2019/09/15/the-conclusion-of-the-activity-of-medical-education-center-to-train-reproductive-health-providers/. Published 2019. Accessed 28 Oct 2019.

[40] OCHA. Border-Crossing Status | HumanitarianResponse. https://www.humanitarianresponse.info/en/operations/stima/border-crossing-status. Accessed 23 July 2019.

[41] Theodorakopoulou E, Goutos I, Mason K, Ghanem AM, Myers S (2019). London calling Gaza: The role of international collaborations in the globalisation of postgraduate burn care education. Scars Burn Heal.

[42] Donaldson RI, Mulligan DA, Nugent K, et al. Using tele-education to train civilian physicians in an area of active conflict: Certifying Iraqi physicians in pediatric advanced life support from the United States. J Pediatr. 2011;159(3). 10.1016/j.jpeds.2011.05.003. 10.1016/j.jpeds.2011.05.003 21722915

[43] Kovacevic P, Dragic S, Kovacevic T (2019). Impact of weekly case-based tele-education on quality of care in a limited resource medical intensive care unit. Crit Care.

[44] UK Aid. Protecting Healthcare in Syria.; 2018. https://assets.publishing.service.gov.uk/media/5ba11d8ae5274a55a85179cd/Research_Report_-_Protection_of_Syrian_Health_Workers__August_2018.pdf.

[45] Barnett-Vanes A, Hassounah S, Shawki M (2016). Impact of conflict on medical education: a cross-sectional survey of students and institutions in Iraq. BMJ Open.

[46] Kerr Winter B, Salamma RM, Qabaja KA (2015). Medical education in Palestine. Med Teach.

[47] Atrache S. Losing Their Last Refuge INSIDE IDLIB’S HUMANITARIAN NIGHTMARE.; 2019.

[48] Percival V, Sondorp E (2010). A case study of health sector reform in Kosovo. Confl Health.

[49] UOSSM. *Syrian Hospitals Surveillance Study*.; 2017. https://www.uossm.nl/upload/jaarverslag-2017.pdf.

[50] WMA Resolution on Task Shifting from the Medical Profession – WMA – The World Medical Association. https://www.wma.net/policies-post/wma-resolution-on-task-shifting-from-the-medical-profession/. Published 2019. Accessed 22 July 2019.

[51] اتحادات الطلبة تشرخ الجامعات السورية وتتحول إلى مراكز اعتقال وتعذيب, أخبــــــار. https://archive.aawsat.com/details.asp?section=4&article=715806&issueno=12487#.XU1HfOhKjb0https://ar.qantara.de/content/قمع-شبيحة-الأسد-لأساتذة. Published 2013. Accessed 15 Oct 2019.

[52] WHO. WHO EMRO | EHA homepage | Emergencies | Entity. http://www.emro.who.int/entity/eha/index.html. Published 2019. Accessed 22 July 2019.

[53] Footer KHA, Clouse E, Rayes D, Sahloul Z, Rubenstein LS (2018). Qualitative accounts from Syrian health professionals regarding violations of the right to health, including the use of chemical weapons, in opposition-held Syria. BMJ Open.

[54] HCiD Resource centre. http://healthcareindanger.org/resource-centre/. Accessed 22 July 2019.

[55] Orcutt M, Rayes D, Tarakji A (2019). International failure in northwest Syria: humanitarian health catastrophe demands action. Lancet (London, Engl).

